# Clinical Features of False-Negative Early Gastric Cancers: A Retrospective Study of Endoscopic Submucosal Dissection Cases

**DOI:** 10.1155/2021/6635704

**Published:** 2021-02-08

**Authors:** Kohei Oka, Naoto Iwai, Takashi Okuda, Tasuku Hara, Yutaka Inada, Toshifumi Tsuji, Toshiyuki Komaki, Junichi Sakagami, Yuji Naito, Keizo Kagawa, Yoshito Itoh

**Affiliations:** ^1^Department of Gastroenterology and Hepatology, Fukuchiyama City Hospital, Fukuchiyama City, Kyoto, Japan; ^2^Department of Molecular Gastroenterology and Hepatology, Graduate School of Medical Science, Kyoto Prefectural University of Medicine, Kyoto, Japan

## Abstract

**Background:**

We frequently encounter early gastric cancer (EGC) that could not be detected in the previous esophagogastroduodenoscopy even if the procedure was annually performed. However, little evidence exists regarding the characteristics of false-negative EGCs. Our aim was to reveal the clinical features of false-negative EGCs.

**Methods:**

We retrospectively reviewed cases of endoscopic submucosal dissection (ESD) for EGCs in Fukuchiyama City Hospital between January 2013 and May 2019. False-negative EGCs were defined as EGCs within 3 years of negative endoscopy. We evaluated the clinical characteristics of false-negative and initially detected EGCs and the difference in the detected and last missed endoscopy in false-negative EGCs. The miss rates of false-negative EGCs were compared between trainees (nonboard-certified endoscopists) and experienced endoscopists (board-certified endoscopists); thereafter, the characteristics of false-negative EGCs missed by trainees were investigated.

**Results:**

Of 219 cases, 119 were classified as false-negative EGCs. False-negative EGCs were characterized as smaller lesions, which presented with normal color or gastritis-like appearance, and were diagnosed after ESD and *H. pylori* eradication (*P* < 0.01). The rate of trainees in the last missed endoscopy was significantly higher than that in the detected endoscopy. The miss rate of false-negative EGC by trainees was higher than that of experienced endoscopists but not significantly different (0.70% vs. 0.57%, *P* = 0.08). The false-negative EGCs missed by trainees were characterized as reddish or well-differentiated lesions, which were located in the lower or lesser curvature of the stomach (*P* < 0.05).

**Conclusion:**

The characteristics of false-negative EGCs were similar to those of *H. pylori*-eradicated EGC. Procedures with shortened examination time and those performed by trainees were risk factors of missing false-negative EGCs. Trainees should pay attention to reddish or well-differentiated EGCs located in the lower or lesser curvature of the stomach.

## 1. Introduction

With the rapid advancement of endoscopic diagnosis, early detection of gastric cancer can contribute to definitive therapy using endoscopic submucosal dissection (ESD) [1]. While annual endoscopic surveillance is recommended after ESD because of a higher incidence rate of metachronous cancers [2], its detection sensitivity to early gastric cancer (EGC) leaves room for improvement [3]. In fact, we frequently encounter EGCs that could not be detected in the previous esophagogastroduodenoscopy (EGD) even if the procedure was performed annually [4, 5]. Thus, we need to decrease the miss rate of EGCs in EGD.

The adenoma detection rate has been recognized as a good quality indicator in colonoscopy [6], while direct evidence for the detection or miss rate of EGCs is limited [3, 7]. Some previous studies have reported the probability of missing advanced gastric cancer [8, 9], while only a few studies have focused on EGC [10]. A previous meta-analysis reported that the miss rate of upper gastrointestinal cancers including esophageal, duodenal, or advanced gastric cancer was 4.6%-25.8% [9]. A Japanese report showed that 75.2% of gastric superficial neoplasia cases were missed in the previous EGD [11]. With regard to the miss rate of EGC, a Korean report addressed that the rate of interval EGC within 2 years was 18.3% [12]. These studies presented some clinical features of false-negative EGC; however, few studies have compared endoscopic features before and at detection of EGC and the miss rates between experienced and trainee endoscopists [4]. Therefore, we aimed to elucidate the clinical features of false-negative EGC considering the endoscopic proficiency level.

## 2. Materials and Methods

### 2.1. Patients

**Figure 1:**
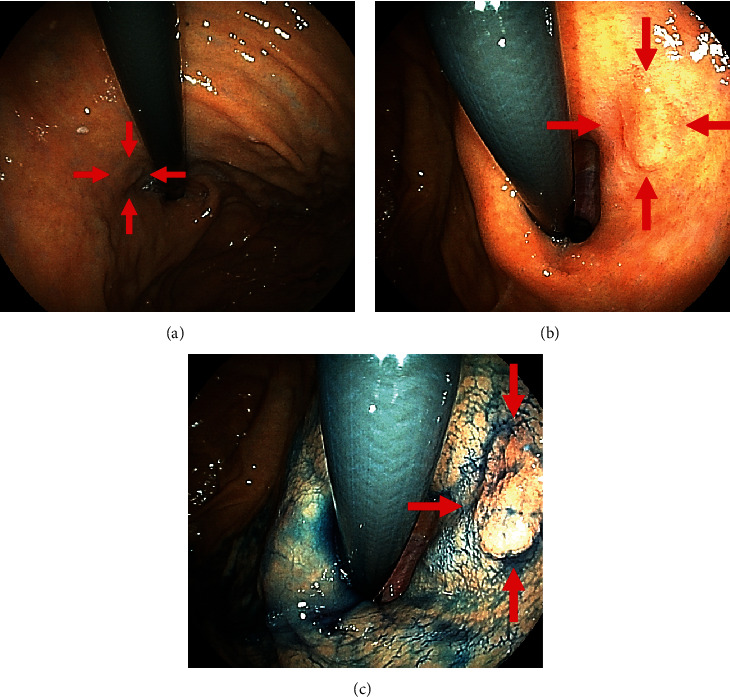
An example of a false-negative EGC. This patient had undergone EGD for an annual medical checkup after *Helicobacter pylori* eradication. (a) Endoscopy 8 months before detection. The cardia was observed in the retroflex view, but the scope was not close to the lesion located in the cardia at the lesser curvature, which can be recognized retrospectively (arrowhead). (b) Endoscopy at the time of detection. A slightly elevated lesion is shown (arrowhead). (c) It is clearly visible by indigo carmine chromoendoscopy. The biopsy specimen reveals well-differentiated adenocarcinoma. EGC: early gastric cancer; EGD: esophagogastroduodenoscopy.

In this study, we retrospectively reviewed data of 375 patients undergoing gastric ESD in Fukuchiyama City Hospital between January 2013 and May 2019. The inclusion criterion for this study was lesions treated with gastric ESD. The exclusion criteria were as follows: (a) uncompleted ESD, (b) EGC diagnosed in other institutions, (c) EGC simultaneously resected with other lesions, (d) pathologically not adenocarcinoma, and (e) no evidence of tumorous tissue in the resected specimen. They were divided into false-negative and initially detected EGCs. False-negative EGCs were defined as missed lesions as previously described [7, 9], despite the fact that EGD was performed at least once within 3 years (+3 months as allowance) prior to the detection of EGC (Figure 1). Initially detected EGCs were defined as lesions detected in patients without EGD in the past 3 years (+3 months as allowance).

First, the clinical characteristics were compared between the false-negative and initially detected EGCs. Second, the characteristics of the detected and last missed endoscopy in false-negative EGCs were investigated. Third, the miss rates between experienced and trainee endoscopists were evaluated using the number of performed EGD and missed EGC: The number of performed EGD was calculated as all EGD between January 2013 and May 2019, and the number of missed EGC was calculated as the sum of missing false-negative EGCs. The procedure time on missed EGCs, which was defined as the duration of all missed endoscopy in false-negative EGCs within 3 years, was also compared. Finally, to analyze the characteristics of false-negative EGCs missed by trainees, the rates of trainees in the detected and last missed endoscopy were compared in terms of tumor location, tumor morphology, color, and pathological differentiation.

### 2.2. Data Collection

**Figure 2:**
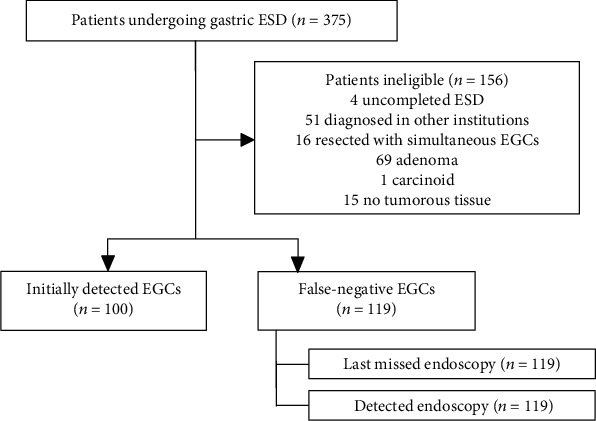
Assignment profile.

Clinical data were collected by chart review (Figure 2). Every endoscopic image was reassessed by the authors referring to the reported findings. A total of 26 endoscopists were classified into trainee and experienced endoscopists. Trainee endoscopists were defined as nonboard-certified fellows of the Japan Gastroenterological Endoscopy Society, while experienced endoscopists were board-certified fellows who had an experience in endoscopy of at least 5 years [13]. Locations of the lesions were categorized into four circumferential parts and three longitudinal parts according to the Japanese classification of gastric carcinoma proposed by the Japanese Gastric Cancer Association [14]. Gastritis-like appearance was defined as a slightly elevated or depressed lesion with similar mucosal patterns to the surrounding noncancerous area [15]. *Helicobacter pylori* (*H. pylori*) infection status was divided into “currently infected,” “past infected,” and “uninfected.” They were determined by serum IgG antibodies or a ^13^C-urea breath test [16, 17]. “Currently infected” patients were defined as those who tested positive for those laboratory findings. “Past infected” patients were defined as those who underwent successful eradication therapy or tested negative for them despite the presence of atrophic gastritis. “Uninfected” was defined as those who tested negative for them in addition to the absence of atrophic gastritis.

### 2.3. Endoscopic Examinations

As preparation for endoscopy, oral dimethicone (Gascon drops; Kissei Pharmaceutical Co., Ltd, Matsumoto, Japan), 1 g of sodium bicarbonate, and 8% lidocaine pump spray (8% Xylocaine Pump Spray; AstraZeneca K.K., Osaka, Japan) were administered. In this study, the endoscopy system used was categorized as having normal, small-diameter, and magnifying endoscopes. Normal endoscopes were GIF-H290, GIF-H260, GIF-Q260, and GIF-260J (Olympus Optical, Tokyo, Japan). Small-diameter endoscopes were GIF-XP290N, GIF-XP260N (Olympus Optical), EG-L580NW7, EG-580NW2, EG-530NW, and EG-530N (Fujifilm Co, Tokyo, Japan). Magnifying endoscopes were GIF-H290Z and GIF-H260Z (Olympus Optical). Every examination of the stomach was routinely performed with white light imaging, besides, indigo carmine chromoendoscopy [18] or image-enhanced endoscopy (narrow-band imaging [19–21], flexible spectral imaging color enhancement [22], blue laser imaging [23], and linked color imaging [24, 25]) was available to augment detection. Some patients underwent midazolam sedation during the procedure upon their request. In addition, topical administration of l-menthol (Minclea; Nihon Pharmaceutical Co., Ltd, Tokyo, Japan) was available for the reduction of gastric peristalsis [26].

### 2.4. Statistical Analysis

Numerical variables were assessed by Welch's *t* test. Categorical variables were assessed by Student's *χ*^2^ test. *P* < 0.05 was considered statistically significant. All statistical analyses were conducted using JMP14.3 (SAS Institute Inc., Cary, NC, USA).

This study was carried out in accordance with the standards of the Declaration of Helsinki, and the protocol was approved by the institutional review board of Fukuchiyama City Hospital (Approval Date: August 22, 2019). The opt-out method was used for obtaining informed consent because of the retrospective design.

## 3. Results

We have reviewed 375 patients undergoing gastric ESD; 156 patients were excluded by the criteria shown in Figure 2. The enrolled 219 patients were categorized into two groups: initially detected EGCs (*n* = 100) and false-negative EGCs (*n* = 119). As a result, the rate of false-negative EGCs was 54.3% (119/219). Of the 26 endoscopists, 65.4% (17/26) were classified into trainee endoscopists.

Table 1 shows the clinical characteristics of the initially detected and false-negative EGCs. Compared with the initially detected EGCs, false-negative EGCs were characterized as smaller lesions (*P* < 0.01), which presented with normal color (*P* < 0.01) or gastritis-like appearance (*P* < 0.01), and were diagnosed after ESD (*P* < 0.01) and *H. pylori* eradication (*P* < 0.01). Patients with false-negative EGCs were significantly asymptomatic and underwent EGD for surveillance or annual medical checkup (*P* < 0.01 and *P* < 0.01, respectively).

Table 2 shows the comparison between the detected and last missed endoscopy in false-negative EGCs. The rate of trainee endoscopists in the last missed endoscopy was significantly higher than that in the detected endoscopy (*P* < 0.05), although endoscope types and use of sedative agents or l-menthol did not contribute to the significant difference (*P* = 0.42, *P* = 0.70, and *P* = 0.52, respectively). Regarding indigo carmine use, duration of examination, numbers of endoscopic pictures obtained, and rate of biopsies, significant differences were found between the detected and last missed endoscopy (*P* < 0.01). The biopsies for the false-negative EGCs were performed on 14 cases (11.8%) in the last missed endoscopy.

Table 3 shows the miss rate of false-negative EGC between experienced and trainees, accompanied by the procedure time of examinations on missed EGCs. The miss rate by trainees was higher than that of experienced endoscopists, but no significant difference was observed (0.70 vs. 0.57%, *P* = 0.08). Trainees took significantly longer procedure time on missed EGCs compared with experienced endoscopists (479 sec vs. 430 sec, *P* < 0.01).

**Figure 3:**
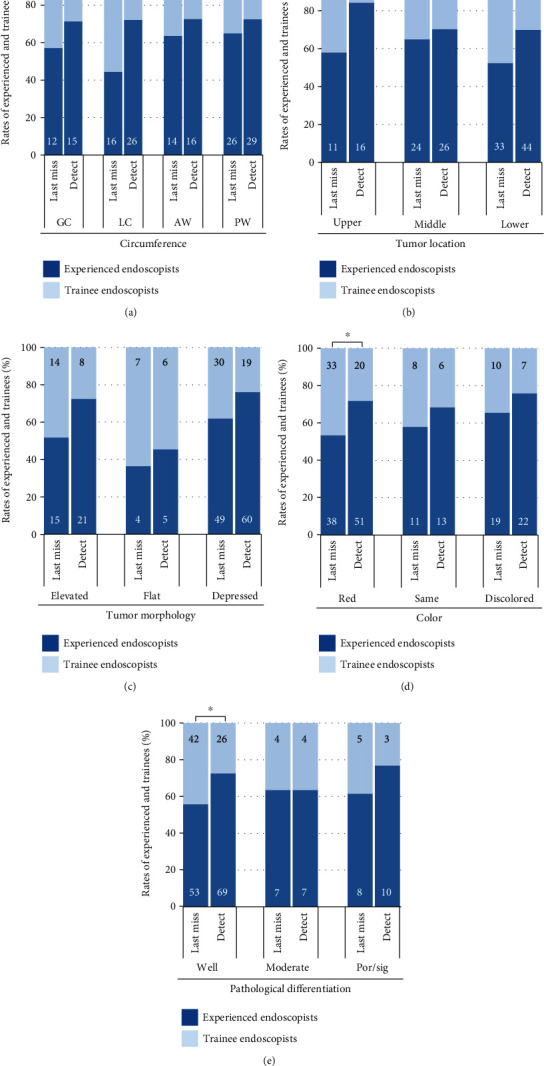
The rates of experienced and trainee endoscopists in the detected and last missed endoscopy of false-negative EGCs regarding (a) circumference, (b) tumor location, (c) tumor morphology, (d) color, and (e) pathological differentiation. The numbers on the graph bars indicate the number of endoscopists in the detected and last missed endoscopy. ^∗^*P* < 0.05, upper: upper stomach; middle: middle stomach; lower: lower stomach; GC: greater curvature; LC: lesser curvature; AW: anterior wall; PW: posterior wall; well: well-differentiated carcinoma; moderate: moderately-differentiated carcinoma; por/sig: poorly-differentiated or signet-ring cell carcinoma.

Figure 3 shows the rates of experienced and trainee endoscopists in the detected and last missed endoscopy of false-negative EGCs regarding tumor location, tumor morphology, color, and pathological differentiation (Figure 3). False-negative EGCs missed by trainees were characterized as reddish or well-differentiated lesions, which were located in the lower or lesser curvature of the stomach (*P* < 0.05).

## 4. Discussion

In this study, we revealed that false-negative EGCs, which were defined as EGCs within 3 years of negative endoscopy, accounted for 54.3% of all EGCs. False-negative EGCs were characterized as smaller lesions, which presented with normal color or gastritis-like appearance, and were diagnosed after ESD and *H. pylori* eradication. The rate of trainee endoscopists in the last missed endoscopy was significantly higher than that in the detected endoscopy. The false-negative EGCs missed by trainees were characterized as reddish or well-differentiated lesions, which were located in the lower or lesser curvature of the stomach. To our knowledge, this is the first study to assess the characteristics of false-negative EGCs missed by trainees.

In this study, we defined false-negative EGCs as missed lesion despite the fact that EGD was performed within 3 years, as previously described [9], although there is no standard definition of false-negative EGCs. Compared with those in previous studies, the rate of false-negative EGCs in our study is relatively higher [9]. It is possibly because false-negative EGCs include interval cancers that developed during the screening interval. Another possible reason is that our institution has a role in education and instruction, because 40% of all EGDs were performed by trainees.

Our study revealed that smaller tumor size and higher rates of patients undergoing EGD for screening or medical checkup were associated with false-negative EGCs, which is consistent with a previous study [12]. Furthermore, we revealed that the diagnoses after ESD and past *H. pylori* infection were associated with false-negative EGCs. The characteristics of “gastritis-like” appearance could indicate influence by *H. pylori* eradication [15, 27]. Smaller tumor is also common after *H*. *pylori* eradication [28]. Our results indicated several similarities between the characteristics of false-negative EGC and those of *H. pylori*-eradicated EGC. Thus, we should pay attention to the possibility of false-negative EGCs in *H. pylori*-eradicated patients.

In comparison between the detected and last missed endoscopy, we found that procedures with shortened examination time and those performed by trainees were risk factors of missing false-negative EGCs, and these findings were consistent with those in previous studies [4, 5]. With regard to the numbers of pictures and the rate of indigo carmine use, it may be explained that EGC detection itself could cause an increase in them.

In this study, we showed that the miss rate by trainees was higher than that of experienced endoscopists, but no significant difference was observed. In addition, we revealed that trainees took significantly longer procedure time on missed EGCs than experienced endoscopists. Although a shorter examination time was reported to be a risk for missing EGCs [5], the miss rate by trainees was higher in spite of a longer examination time. Collectively, our study suggested that the proficiency level of endoscopists may be related to missing the false-negative EGC.

Finally, we have focused on the characteristics of false-negative EGCs missed by trainees. As regards tumor location, trainees missed the lesions in the lower or lesser curvature of the stomach compared with experienced endoscopists. We speculated that insufficient air insufflation and poor upside angulation were attributed to a high miss rate in the lesser curvature, and poor observation due to gastric peristalsis might result in missing EGCs in the lower stomach [29]. EGCs in the posterior wall are difficult to detect because of the tangential endoscopic vision to the posterior wall [30]; however, no significant difference in detection rates was noted. It could be due to a fact that even experienced endoscopists had difficulty in detecting lesions on the posterior wall [30]. As regards tumor color, we assumed that reddish EGCs, which are often associated with well-differentiated tumor [31], may be a good target for experienced endoscopists, who understand the typical characteristics of EGC.

Our study has several limitations. First, this is a retrospective single-center study, and cases not indicated for ESD were excluded. Hence, false-negative invasive gastric cancers that were treated with gastrectomy or chemotherapy were not analyzed. Second, the cumulative experience of each endoscopist was not evaluated; therefore, the result does not account for the learning curve of trainee endoscopists. Finally, in this study, false-negative EGCs are possibly including interval cancers which were developed during follow-up time after negative endoscopy, because it is difficult to distinguish between real false-negative cancer and interval cancer. Many false-negative EGCs were actually difficult to point out in the previous endoscopic images because they often showed slight changes or, in some cases, they were unsatisfactory for assessment.

## 5. Conclusion

We have revealed that the characteristics of false-negative EGC were similar to those of *H. pylori*-eradicated EGC. In addition, we found that procedures with shortened examination time and those performed by trainees were risk factors of missing false-negative EGCs. Compared with experienced endoscopists, trainees should pay attention to reddish or well-differentiated EGCs located in the lower or lesser curvature of the stomach.

## Figures and Tables

**Table 1:** Clinical characteristics in the initially detected and false-negative EGCs.

	Initially detected EGCs (*n* = 100)		False-negative EGCs (*n* = 119)		*P* value
Age (years)	71	[61–79]	72	[63–79]	0.61
Men	73	(73.0%)	86	(72.3%)	0.90
Previous ESD/EMR	1	(1.0%)	34	(28.6%)	<0.01
Symptomatic	30	(30.0%)	12	(10.1%)	<0.01
Atrophic gastritis					0.38
Opened type	87	(87.0%)	108	(90.8%)	
None or closed type	13	(13.0%)	11	(9.2%)	
*Helicobacter pylori* status					<0.01
Currently infected	75	(75.0%)	50	(42.0%)	
Past infected	18	(18.0%)	64	(53.8%)	
Uninfected	7	(7.0%)	5	(4.2%)	
Endoscopist					0.59
Experienced	69	(69.0%)	86	(72.3%)	
Trainee	31	(31.0%)	33	(27.7%)	
Purpose					<0.01
Screening	78	(78.0%)	43	(36.1%)	
Surveillance	1	(1.0%)	37	(31.1%)	
Annual medical checkup	21	(21.0%)	39	(32.8%)	
Tumor size (mm)	15	[9–23]	9	[5–13]	<0.01
Depth					0.09
M	90	(90.0%)	115	(96.6%)	
sm1	2	(2.0%)	0	(0%)	
sm2 or deeper	8	(8.0%)	4	(3.4%)	
Pathological differentiation					0.19
Well or moderate	83	(83.0%)	106	(89.1%)	
Poorly	17	(17.0%)	13	(10.9%)	
Location					0.82
Upper	13	(13.0%)	19	(16.0%)	
Middle	33	(33.0%)	37	(31.1%)	
Lower	54	(54.0%)	63	(52.9%)	
Circumference					0.51
Anterior wall	23	(23.0%)	22	(18.5%)	
Posterior wall	25	(25.0%)	40	(33.6%)	
Lesser curvature	35	(35.0%)	36	(30.3%)	
Greater curvature	17	(17.0%)	21	(17.7%)	
Color					<0.01
Reddish	55	(55.0%)	71	(59.7%)	
Discolored	40	(40.0%)	29	(24.4%)	
Normal	5	(5.0%)	19	(16.0%)	
Tumor morphology					0.33
Elevated	33	(33.0%)	29	(24.4%)	
Flat	9	(9.0%)	11	(9.2%)	
Depressed	57	(58.0%)	79	(66.4%)	
Erosion					0.86
Present	45	(45.0%)	55	(46.2%)	
Absent	55	(55.0%)	64	(53.8%)	
Gastritis-like appearance					<0.01
Present	15	(15.0%)	42	(35.3%)	
Absent	85	(85.0%)	77	(64.7%)	
Spontaneous bleeding					0.72
Present	16	(16.0%)	17	(14.3%)	
Absent	84	(84.0%)	102	(85.7%)	

Data are presented as median (IQR) or *n* (%). EGC: early gastric cancer; ESD: endoscopic submucosal dissection; EMR: endoscopic mucosal resection.

**Table 2:** Comparison between the detected and last missed endoscopy.

	Detected endoscopy (*n* = 119)		Last missed endoscopy (*n* = 119)		*P* value
Symptom					0.65
Symptomatic	12	(10.1%)	10	(8.4%)	
Asymptomatic	107	(89.9%)	109	(91.6%)	
Purpose					0.81
Screening	43	(36.1%)	43	(36.1%)	
Surveillance	37	(31.1%)	33	(27.7%)	
Annual medical checkup	39	(33.0%)	43	(36.1%)	
Endoscopist					<0.05
Experienced	86	(72.3%)	68	(57.1%)	
Trainee	33	(27.7%)	51	(42.9%)	
Endoscope					0.42
Normal	73	(61.3%)	74	(62.2%)	
Small-diameter	35	(29.4%)	39	(32.8%)	
Magnifying	11	(9.2%)	6	(5.0%)	
Duration (sec)	519	[427–618]	433	[371–531]	<0.01
Numbers of pictures	72	[65-83]	66	[58-72]	<0.01
Sedative drug use	3	(2.5%)	4	(3.4%)	0.70
L-menthol use	6	(5.0%)	4	(3.4%)	0.52
Indigo carmine use	118	(99.2%)	102	(85.7%)	<0.01
Biopsies	116	(97.5%)	56	(47.1%)	<0.01

Data are presented as median (IQR) or *n* (%).

**Table 3:** Miss rate and procedure time on missed EGCs between experienced and trainee endoscopists.

	Performed EGD	Missed EGCs	Miss rate	Procedure time on missed EGCs
Experienced	28219	165	0.57%	430 [371-530]
Trainees	19110	134	0.70%	479 [387-615]
Total	47329	299	0.62%	444 [374-551]

Data are presented as median (IQR) or *n*. EGC: early gastric cancer; EGD: esophagogastroduodenoscopy.

## Data Availability

The patient data used to support the findings of this study are available from the corresponding author upon request.
